# Supplementary material for: Predicting visual function by interpreting a neuronal wiring diagram
Source: Nature. 2024 Oct 2;634(8032):113–23. doi: 10.1038/s41586-024-07953-5 (PMC11446822; doi:10.1038/s41586-024-07953-5)
Supplement: Supplementary file 6 — CRF and ERF predictions for individual TmY4 and TmY9 cells. Analogous to Supplementary Data 3, but for TmY target types. Shown are the top four monosynaptic pathways, the strongest pathway passing through each of the top ten intermediary types (ranking from Extended Data Fig. 7), and the trisynaptic pathway Tm1–TmY–Dm3–TmY (see the section entitled Prediction of spatial normalization). [file 41586_2024_7953_MOESM6_ESM.zip › DataS4/TmY9q/720575940624964452.pdf]

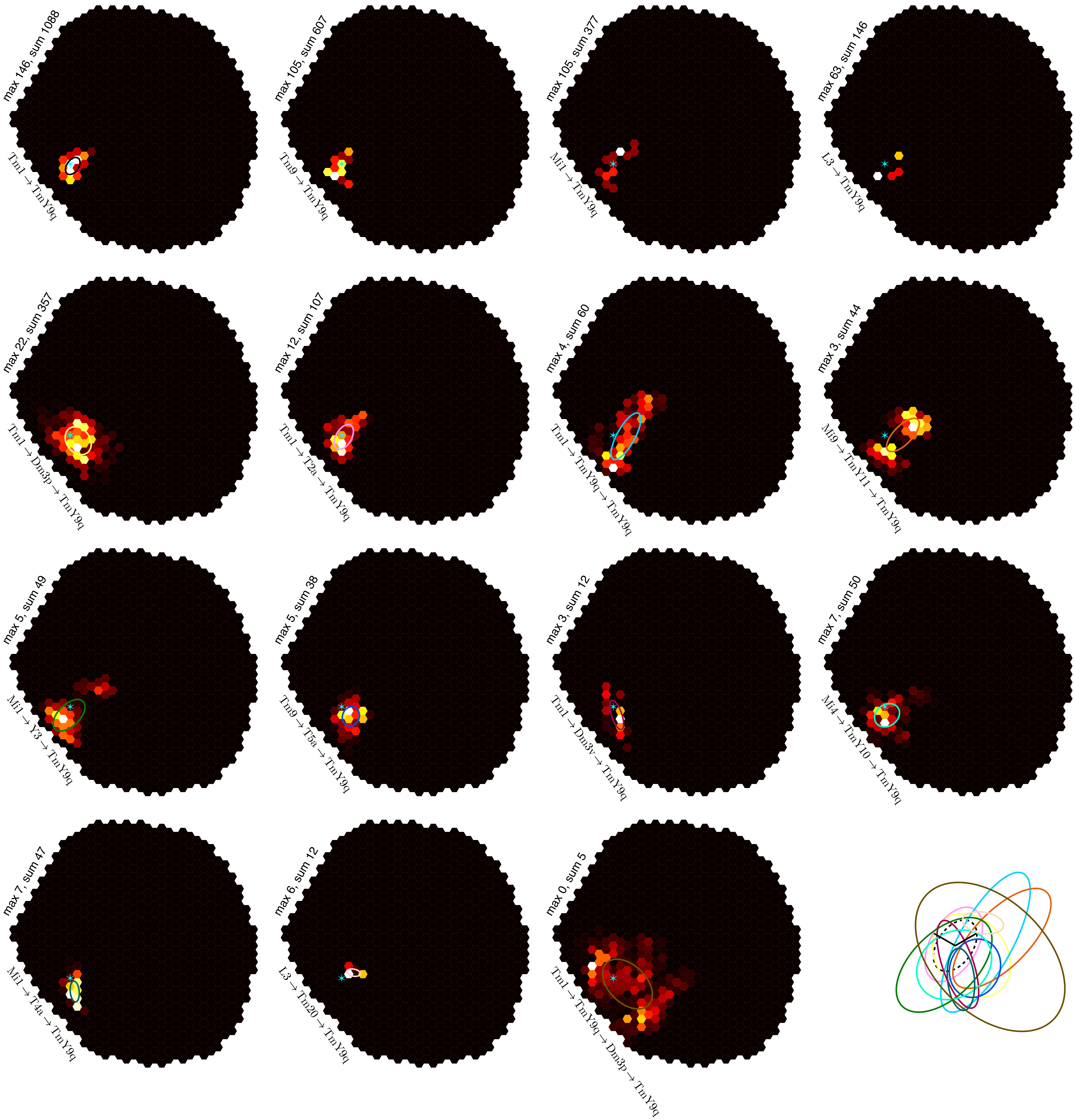
max 146, sum 1088
Tm1 → TmY9q
max 105, sum 607
Tm9 → TmY9q
max 105, sum 377
Mi1 → TmY9q
max 63, sum 146
L3 → TmY9q
max 22, sum 357
Tm1 → Dm3p → TmY9q
max 12, sum 107
Tm1 → T2a → TmY9q
max 4, sum 60
Tm1 → TmY9q → TmY9q
max 3, sum 44
Mi9 → TmY11 → TmY9q
max 5, sum 49
Mi1 → Y3 → TmY9q
max 5, sum 38
Tm9 → T5a → TmY9q
max 3, sum 12
Tm1 → Dm3v → TmY9q
max 7, sum 50
Mi4 → TmY10 → TmY9q
max 7, sum 47
Mi1 → T4a → TmY9q
max 6, sum 12
L3 → Tm20 → TmY9q
max 0, sum 5
Tm1 → TmY9q → Dm3p → TmY9q
